# A low-latency deep learning framework for volcanic ash cloud nowcasting using geostationary satellite imagery

**DOI:** 10.1038/s41598-026-42230-7

**Published:** 2026-03-22

**Authors:** Décio Alves, Marko Radeta, Fábio Mendonça, Lucas Pereira, Fernando Morgado-Dias

**Affiliations:** 1 University of Madeira, Campus Universitário da Penteada, 9020-105 Funchal, Portugal; 2 Interactive Technologies Institute (ITI/LARSyS and ARDITI), Edif. Madeira Tecnopolo, Caminho da Penteada piso -2, 9020-105 Funchal, Portugal; 3 Wave Labs / MARE - Marine and Environmental Sciences Centre / ARNET - Aquatic Research Network, Agência Regional para o Desenvolvimento da Investigação Tecnologia e Inovação (ARDITI), Funchal, Portugal; 4 Department of Astronomy, Faculty of Mathematics, University of Belgrade, Belgrade, Serbia; 5 Instituto Superior Técnico, University of Lisbon, Lisbon, Portugal

**Keywords:** Environmental sciences, Mathematics and computing, Natural hazards, Solid Earth sciences

## Abstract

Rapid assessment of hazardous aerosol dispersion is critical for emergency response, yet operational dispersion workflows can exhibit end-to-end latency that is incompatible with the first minutes of decision-making. This study develops and validates a deep learning approach for near-real-time nowcasting of *volcanic ash* dispersion from geostationary observations. The model was trained on an archive of volcanic ash satellite imagery from EUMETSAT’s SEVIRI instrument (Ash RGB composite) and achieved a structural similarity index of 0.88 for 15-minute next-frame forecasts. The complete edge workflow, including data download and inference, runs in under five seconds on an NVIDIA Jetson AGX Orin. To illustrate how the same nowcasting pipeline can be used for hypothetical scenario exploration across particulate sources, a pixel-based event-injection algorithm is introduced to overlay synthetic plumes of varying sizes into real-time satellite frames before inference. Scenario demonstrations parameterized by nuclear-yield-inspired sizes (10 kt to 100 Mt) are presented at urban (Paris, London, Berlin), national (Iberian Peninsula), and continental (Europe-wide) scales. These scenario outputs are intended as illustrative, low-latency visualizations of kinematic transport patterns in the SEVIRI observation space, as validated predictions of nuclear plume morphology. The primary contribution is a fast, low-cost volcanic ash nowcasting system, complemented by a generalizable injection framework for rapid scenario visualization on edge computing.

## Introduction

Volcanic eruptions, nuclear fallout, and radiological releases inject hazardous aerosols into the atmosphere, threatening aviation, infrastructure, public health, global security, and climate^1^. As an example, the volcanic ash clouds from the 2010 Eyjafjallajökull event caused a week-long shutdown of European airspace, with an estimated revenue loss of USD 1.7 billion within 6 days from the event^2^. Such particulates may also travel large distances, as recorded in nuclear fallout from nuclear plant disasters, spreading to whole countries^3^ and other continents within 3–4 days^4^. Furthermore, inhalation of such particulates, for example crystalline silica-rich ash, can cause long-term respiratory consequences^5^, while direct exposure to radioactive dust can provoke acute radiation syndrome, resulting in cataracts, skin injuries, and sexual dysfunction^6^.

Another important aspect is the adverse effects of artificial radiation belts and trapped energetic particles produced by high-yield nuclear tests such as Starfish Prime (1,500 kt), Castle Bravo (15,000 kt), and Tsar Bomba (50,000 kt). Their long-term impacts remain incompletely characterized^7^. Because direct fallout observations are limited, studies often rely on computer models, and the lack of comprehensive fallout datasets remains a challenge^8^. This is also the case when forecasting dispersion of hazardous particulates, which is relevant for informing emergency centers and populations about exposure and routing^9^. Models such as the Hybrid Single-Particle Lagrangian Integrated Trajectory (HYSPLIT) by the National Weather Service can forecast volcanic ash^10^ and nuclear fallout^11^. Such models usually perform physically based atmospheric transport and dispersion modeling by computing atmospheric trajectories and simulations using either puff or Lagrangian particles^12^.

However, models such as HYSPLIT typically rely heavily on forecasts from numerical weather prediction models, which might require substantial preprocessing (such as conversion of GRIB data to ARL format) and automated scripting for scheduled forecasts. While effective, this process is computationally intensive and time-consuming^13^, and becomes highly dependent on the accuracy and resolution of the underlying models. Bowman et al.^14^ noted that Lagrangian dispersion models can only improve if the mesoscale models they rely on become more detailed in spatial and temporal resolutions. In contrast, machine learning approaches can provide a data-driven alternative that learns atmospheric patterns from historical and real-time data^15^, enabling rapid forecasts without explicit physical modeling or heavy computational infrastructure. A further practical advantage documented in the literature is computational efficiency, where AI-based forecast models can produce predictions in a very short time compared to traditional numerical systems, which is critical for rapid-response operations^16^.

Beyond offline Lagrangian dispersion with prescribed meteorology, online-coupled systems jointly resolve meteorology and scavenging and represent the current state of the art for radionuclide transport. Fang et al.^17^ integrated 25 combinations of in- and below-cloud wet-scavenging schemes for ^137^Cs within WRF-Chem and found that coupling with cloud microphysics improved precipitation, cumulative deposition, and atmospheric concentrations relative to below-cloud-only baselines. These results underscore the benefits of online coupling for process fidelity. The present study targets a different operational niche: immediate, low-latency nowcasting from geostationary observations.

It was shown that numerical simulations can lead to robust models to estimate atmospheric ash dispersal. For instance, the hybrid Lagrangian VOL-CALPUFF model coupled a one-dimensional plume module to kilometre-scale puff tracking^18^, while the fully Eulerian FALL3D model represents atmospheric passive transport and deposition using 3D advection–diffusion–sedimentation with high-performance-computing scalability^19,20^, quantifying confidence intervals in minutes^21^. Coupling the same model with an ensemble transform Kalman filter reduced concentration errors by more than half in controlled tests^22^, with more recent versions establishing benchmark suites spanning ash and radionuclides^20^.

Although machine learning techniques have been applied to the detection of volcanic clouds and ash^23^, to the best of the authors’ knowledge these methods have not yet been used to predict atmospheric ash dispersal. Nonetheless, such an approach is likely to be effective, as machine learning has demonstrated success in forecasting broader atmospheric patterns^15^.

In this work, volcanic ash is used as the validated target phenomenon for nowcasting from SEVIRI imagery. Hazard motivation includes radiological releases because atmospheric transport shares common advection and turbulent dispersion physics across fine particulate types. Volcanic ash can be an analogue for the post-injection phase, where micron-scale particle properties and ambient meteorology govern advection, turbulent dispersion, and deposition. Nuclear fallout frequently includes silicate glass particles and cesium-bearing microparticles with sizes comparable to fine volcanic ash, supporting the analogy for aspects of atmospheric transport and deposition^24–27^, and similarly affected by wind patterns and atmospheric conditions^28^. At the same time, cesium-137 can exist in soluble forms or attached to multi-mode aerosols, exhibiting complex in-cloud and below-cloud scavenging behaviors during precipitation that depend on particle size, number concentration, hygroscopicity, and cloud microphysics^17,29,30^. The nowcasting framework presented here does not explicitly represent solubility-dependent activation or size-resolved wet scavenging. Outputs should therefore be interpreted as kinematic transport and bulk deposition *proxies* in the SEVIRI observation space, with potential biases in precipitating scenes where aqueous scavenging dominates.

On the same line, multi-purpose systems originally built for air-quality management, such as NAME and DERMA, have been adapted for nuclear applications, exploiting the common fluid-dynamic framework that governs both silicate and radionuclide particulates^31,32^. Monitoring centers such as the London Volcanic Ash Advisory Center (VAAC) have upgraded models with improved microphysics and satellite validation to sustain dependable service levels^33^. Progress in these fields has been chronicled in reviews of tephra transport^34^ and real-time air-quality forecasting^35^.

Still, forecasting dispersion is highly sensitive to uncertainties, typically caused by source parameters including eruption rate, cloud height, radionuclide inventory, and wind field errors^1^. Because near-real-time volcanic ash location can be obtained via satellite remote sensing such as SEVIRI^36^, limited efforts exist in nowcasting of such particulates^37^.

Fukushima-era fallout datasets (e.g., hourly $^{134/137}$Cs SPM and gridded deposition products) are publicly available^38–40^, but they do not provide SEVIRI-like 15-minute, Europe-wide satellite image sequences in the 2020–2025 period needed to directly supervise geostationary next-frame prediction. In this sense, the present study validates on volcanic ash observations, and uses radiological scenarios only as demonstrations of a general event-injection workflow.

Since time is of utmost importance for emergency response, this work focuses on near-real-time nowcasting^15^ (forecasting within 2 hours) of volcanic ash dispersion. Specifically, this work uses a spatio-temporal deep neural network model trained on an observational archive of ash dispersion imagery, enabling low-latency nowcasts on edge computing.

To demonstrate how the same pipeline can support rapid scenario visualization, this work also implements a pixel-based event-injection algorithm to overlay synthetic particulate sources (parameterized by yield-inspired sizes between 10 and 100,000 kt) into the most recent SEVIRI frame before inference. This approach is conceptually similar to introducing a source term for a dispersion model, but it operates purely in the image domain to create scenario inputs for the nowcasting model. These scenario demonstrations are complementary to traditional numerical solvers and are intended as low-latency, observation-space visualizations that can be updated as new SEVIRI imagery arrives.

Process-resolving radionuclide dispersion systems at regional and urban scales explicitly represent advection, turbulence, dry and wet deposition, and size-resolved scavenging, achieving high skill when driven by kilometer-scale meteorology and calibrated source terms^41–43^. The objective of this work is complementary: a geostationary-observation nowcast for the first 0–2 h after a visible particulate release that delivers sub-5 s end-to-end latency on edge hardware, without requiring numerical weather prediction ingest, source-term inversion, or site-specific urban morphology.

This work is structured as follows. Section 2 outlines the methodology, detailing the geostationary satellite data, the development and optimization of the deep learning model, the event-injection framework for scenario inputs, and the metrics used for evaluation. Section 3 presents the performance of the model and demonstrates scenario visualizations at urban, country, and continental scales. Finally, Section 4 concludes the paper by summarizing the key findings, discussing limitations, and outlining future work.

## Methods

### Geostationary ash–cloud imagery

This study utilizes the Ash Red, Green, and Blue (RGB) composite produced by SEVIRI on Meteosat Second Generation. Cropped scenes were obtained from the EUMETSAT Web Map Service endpoint with the layer msg_fes:rgb_ash. Tiles were requested every 15 minutes from 1 September 2020 to 1 April 2025, restricted to the bounding box $34^{\circ }\textrm{N}$–$72^{\circ }\textrm{N}$, $11^{\circ }\textrm{W}$–$35^{\circ }\textrm{E}$ in the EPSG:4326 coordinate reference system, and returned as 256 $\times$ 256 pixel TIFF images that preserve the native approximately 3 km nadir sampling of SEVIRI. In total, 168,192 images were collected over the study period. This archive forms the raw satellite input for subsequent machine learning training and evaluation^44,45^. Open nuclear-fallout datasets from Fukushima (atmospheric concentrations and deposition) exist^38–40^, but they are event-limited and lack geostationary RGB imagery at 15-minute cadence over the European domain, so they cannot serve as direct supervised targets for SEVIRI-based next-frame prediction.

**Figure 1:**
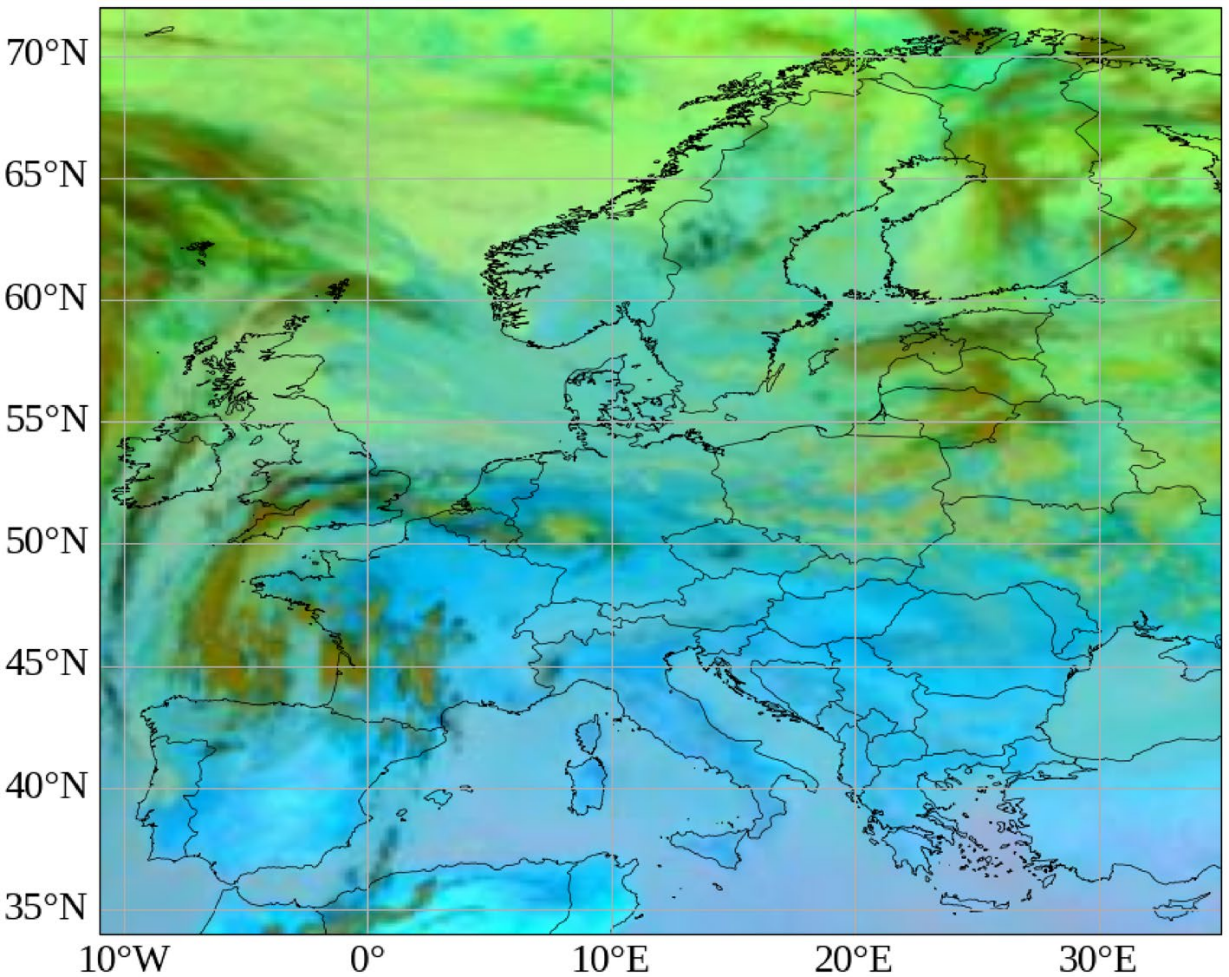
Ash RGB composite from SEVIRI on Meteosat Second Generation, 25 June 2025 at 14:50 UTC (EUMETSAT). Maps generated using Python 3.1 (https://www.python.org/) with OpenStreetMap contributor data (https://www.openstreetmap.org).

Figure 1 shows an example of the Ash RGB composite acquired on 25 June 2025 at 14:50 UTC from EUMETSAT.

### Machine learning nowcasting model

A deep neural network was employed to predict short-term ash cloud evolution from sequences of four consecutive images, representing a 45-minute history at SEVIRI’s 15-minute cadence. The sequence length of four frames was adopted to balance temporal context and computational latency, consistent with previous satellite-based nowcasting studies^46^. The architecture consists of a sequence-to-one configuration, with an encoder comprising two Convolutional Long Short-Term Memory (ConvLSTM) layers and a decoder based on a two-dimensional convolution applied to the final hidden state to produce the output image. The ConvLSTM layers used Rectified Linear Unit (ReLU) activations $f(x) = \max (0, x)$, while the output convolution layer used 3 filters and applied a sigmoid activation $f(x) = \frac{1}{1 + e^{-x}}$ to reconstruct the predicted next-frame image. This frame can be recursively fed to the model, allowing predictions for multiple future time steps in an autoregressive manner. This enables 15-minute nowcasts that can be recursively advanced to longer forecast windows.

Formally, each ConvLSTM cell updates its hidden state according to:

1 $$\begin{aligned} i_t = \sigma \bigl (W_{xi} * X_t + W_{hi} * H_{t-1} + W_{ci} \odot C_{t-1} + b_i \bigr ), \end{aligned}$$

2 $$\begin{aligned} f_t = \sigma \bigl (W_{xf} * X_t + W_{hf} * H_{t-1} + W_{cf} \odot C_{t-1} + b_f \bigr ), \end{aligned}$$

3 $$\begin{aligned} C_t = f_t \odot C_{t-1} \;+\; i_t \odot \Phi \bigl (W_{xc} * X_t + W_{hc} * H_{t-1} + b_c \bigr ), \end{aligned}$$

4 $$\begin{aligned} o_t = \sigma \bigl (W_{xo} * X_t + W_{ho} * H_{t-1} + W_{co} \odot C_t + b_o \bigr ), \end{aligned}$$

5 $$\begin{aligned} H_t = o_t \odot \Phi (C_t), \end{aligned}$$

where $X_t$ is the input at time step *t*, $H_t$ is the hidden state, $C_t$ is the cell state, *W* are learnable parameters, $\sigma$ denotes the recurrent activation function (sigmoid), $\Phi$ is the activation function (ReLU was used instead of the standard hyperbolic tangent to integrate with modern optimizers and hardware-accelerated training pipelines; although ReLU outputs are unbounded, subsequent normalization layers and the final sigmoid activation ensure predictions remain within valid pixel ranges), $*$ indicates convolution, and $\odot$ denotes element-wise multiplication. The two-dimensional convolution layer operates without temporal memory or gating mechanisms, thus its operations are expressed as:

6 $$\begin{aligned} Y = \sigma (W * X + b), \end{aligned}$$

where *Y* is the output feature map, *W* represents the convolutional weights, *X* is the input, and *b* is the bias term.

The architecture was based on the concept proposed by Desai et al.^47^ for next-frame prediction using ConvLSTM, and the architecture discussed by Naz et al.^48^ (a convolution layer sequence, followed by two ConvLSTM layers, followed by a convolution layer), where it was concluded that ConvLSTM layers facilitate the model’s ability to analyze spatiotemporal patterns and improve weather predictions. The proposed architecture follows these concepts. It processes input sequences with shape (4, 256, 256, 3), representing four frames of $256\times 256$ pixels with three channels (RGB). The first ConvLSTM2D layer applies *K* convolutional filters of size $F\times F$ to generate feature maps with output shape (4, 256, 256, *K*), preserving temporal length and spatial dimensions while expanding channel depth. A batch normalization layer normalizes feature maps without altering dimensionality using $f(x) = \gamma \cdot \frac{x - \mu }{\sqrt{\nu + \epsilon }} + \beta$, where $\mu$ and $\nu$ are the batch mean and variance, $\gamma$ and $\beta$ are learnable parameters, and $\epsilon$ is a small constant. The second ConvLSTM2D layer applies another set of *K* filters of size $F\times F$, reducing the temporal dimension by outputting only the final hidden state, producing (256, 256, *K*). A second batch normalization layer normalizes these features. Finally, the convolution layer applies 3 filters of size $3\times 3$ to map the *K*-channel representation back to (256, 256, 3), corresponding to the predicted next-frame image.

Therefore, the encoder–decoder architecture operates in the temporal domain, where the encoder processes a sequence of four input frames to extract temporal dynamics into a compressed representation, and the decoder reconstructs this representation into a single predicted frame. Specifically, the ConvLSTM layers function as the temporal encoder by sequentially processing the input time series and encoding temporal patterns into the final hidden state. The last convolutional layer serves as the decoder, mapping the temporally encoded hidden state back to the spatial domain for next-frame prediction.

To improve predictive capability, a hyperparameter grid search optimization was performed to optimize the choice for *K* and *F*. The search explored combinations of the number of filters (32, 64, or 128) and the size of the convolutional kernel (1, 3, or 5), selecting the configuration that yielded the lowest validation error. These hyperparameter ranges were chosen based on commonly adopted values in convolutional architectures for spatiotemporal modeling, providing a trade-off between model expressiveness and computational efficiency while mitigating the risk of overfitting. Similar grid-search limits have been applied in previous ConvLSTM-based nowcasting studies, including Alves et al.^46^, where three values per parameter were sufficient to identify optimal configurations without excessive computational cost. All SEVIRI Ash-RGB composites were converted to $256\times 256$ pixel JPEGs, normalized to the [0, 1] range, and used without augmentation or histogram equalization. Clear-sky frames were retained to maintain temporal continuity and allow the model to learn both ash-present and ash-free conditions. The model was trained using the Mean Squared Error loss and the Adam optimizer (learning rate $1\times 10^{-3}$, $\beta _1=0.9$, $\beta _2=0.999$, $\epsilon =10^{-7}$), with a batch size of 2 and a maximum of 50 epochs, applying early stopping after ten epochs without improvement. Data loading employed batch prefetching and GPU memory growth to maintain stable memory usage. Model development and validation employed strictly time-based data splits, reserving the last ten days of the archive for holdout testing and allocating 10% of the remaining training data for validation. All training and inference were performed on an NVIDIA Jetson AGX Orin (64 GB) using Python 3 and TensorFlow 2.

All training and inference procedures were conducted on an NVIDIA Jetson AGX Orin 64 GB, a high-performance edge computing platform optimized for demanding computational workloads. To evaluate system efficiency, timing metrics were recorded for both data acquisition and inference. The download time for four satellite image frames was measured at 3.289 seconds. The inference process, consisting of eight sequential steps, required a total of 1.708 seconds, with individual step times ranging from 0.115 to 0.809 seconds. The processing workflow, encompassing both data acquisition and inference, sustained a total pipeline latency of under 5 seconds.

### Scenario size parameterization

To parameterize the size of synthetic scenario injections, a practical scaling relation was used to map a nominal “yield” variable into an approximate physical radius. In the nuclear-effects literature, power-law relationships between yield and thermal damage radius have long been used as operational approximations^49^, reflecting that thermal fluence decreases approximately as an inverse square of distance while scaling sub-linearly with explosive yield due to atmospheric attenuation and energy absorption. Here, the second-degree burn radius is used only as a convenient, well-tabulated size proxy to define relative injection extents for scenario visualization. The radius is estimated as

7 $$\begin{aligned} R_{2^\circ }\;[\textrm{km}] \;=\; 0.9\,W^{0.41}, \end{aligned}$$

where $R_{2^\circ }$ is the radius in kilometers and *W* is the nominal yield in kilotons of TNT. The constants in Eq. 7 were obtained by fitting the well-documented 10 kt Hiroshima datum of 2.3 km^50^ and the 14–16 km range measured for a 1 Mt air-burst test^51^. Table 1 lists corresponding radii for representative yields. These values assume a clear atmosphere and an optimum height of burst; heavy cloud, haze, or shielding by terrain or structures will shorten the radii. In this work, these radii support a consistent size ladder for synthetic injections in the image domain.

**Table 1:** Representative nominal yields used for scenario parameterization, with classifications and approximate second-degree burn radii (Eq. 7).

Yield (kt)	Description	Radius (km)
10	Sub-Hiroshima yield (e.g., small fission device)	2.3
15	Hiroshima-class (e.g., Little Boy, 1945)	2.7
50	Intermediate tactical nuke (e.g., W54 max yield)	4.5
100	Standard tactical warhead (e.g., B61 Mod 3, $\sim$170 kt max)	5.9
500	Small strategic nuke (e.g., W88 lower estimate)	11.5
1,000	Strategic thermonuclear warhead (e.g., B83, full yield)	15.3
1,400	Starfish Prime	17.5
10,000	High-yield thermonuclear device (e.g., theoretical)	39.3
15,000	Castle Bravo	46.4
50,000	Tsar Bomba	76.0
100,000	Hypothetical high-yield device	101.0

The spatial resolution of each downloaded frame was estimated from the specified bounding box and image dimensions. The latitude span was $38^\circ$ ($34^\circ$ N to $72^\circ$ N), corresponding to approximately $38 \times 111.32 = 4{,}231$ km in the north–south direction. The longitude span was $46^\circ$ ($11^\circ$ W to $35^\circ$ E), which at the mean latitude of $53^\circ$ N corresponds to $46 \times \bigl (111.32 \times \cos (53^\circ )\bigr ) \approx 3{,}082$ km in the east–west direction. Dividing by the image resolution of $256 \times 256$ pixels yields approximate ground sampling distances of 12 km per pixel horizontally and 16.5 km per pixel vertically. These estimates provide a reference scale for scenario injection sizing and for interpreting predicted plume extents.

### Model optimization and evaluation metrics

Model hyperparameter optimization was performed using a grid search (over combinations of the number of filters and the convolution kernel size). For this optimization stage, the criterion used to rank configurations was the Mean Squared Error (MSE) evaluated on the validation dataset. The MSE quantifies the average squared discrepancy between predicted and observed pixel intensities, and is defined as

8 $$\begin{aligned} \textrm{MSE} = \frac{1}{N} \sum _{i=1}^N \bigl ( \hat{y}_i - y_i \bigr )^2, \end{aligned}$$

where $N$ denotes the total number of pixels, $\hat{y}_i$ is the predicted value of pixel $i$, and $y_i$ is the corresponding ground-truth value.

Furthermore, model performance was assessed considering five evaluation metrics computed on the holdout test dataset as well as on operational scenario experiments. In addition to MSE, the Mean Absolute Error (MAE) was also calculated as

9 $$\begin{aligned} \textrm{MAE} = \frac{1}{N}\sum _{i=1}^N \bigl | \hat{y}_i - y_i \bigr |, \end{aligned}$$

where $|\cdot |$ denotes the absolute value.

The Root Mean Squared Error (RMSE) was also computed to express the error in the same units as the normalized image intensity and is defined as

10 $$\begin{aligned} \textrm{RMSE} = \sqrt{\frac{1}{\textrm{N}} \sum _{i=1}^{\textrm{N}} \left( \hat{\textrm{y}}_{\textrm{i}}- {\textrm{y}}_{\textrm{i}} \right) ^2}. \end{aligned}$$

Peak Signal-to-Noise Ratio (PSNR) was determined, reflecting the ratio of maximum signal power to noise power, expressed in decibels, and was computed according to

11 $$\begin{aligned} \textrm{PSNR} = 10 \log _{10}\Bigl (\frac{L^2}{{\frac{1}{\textrm{N}} \sum _{i=1}^N \bigl ( \hat{\textrm{y}}_{\textrm{i}} - {\textrm{y}}_{\textrm{i}} \bigr )^2}}\Bigr ), \end{aligned}$$

where $L$ is the maximum possible pixel value, here $L=1$.

Lastly, the Structural Similarity Index (SSIM) was estimated as it quantifies perceptual similarity by incorporating luminance, contrast, and structural comparisons as

12 $$\begin{aligned} \textrm{SSIM}(x,y) = \frac{\bigl (2\mu _x \mu _y + C_1\bigr )\bigl (2\sigma _{xy}+C_2\bigr )}{\bigl (\mu _x^2+\mu _y^2+C_1\bigr )\bigl (\sigma _x^2+\sigma _y^2+C_2\bigr )}, \end{aligned}$$

where $\mu _x$ and $\mu _y$ are the means of the predicted and reference images, $\sigma _x^2$ and $\sigma _y^2$ are the variances, $\sigma _{xy}$ is the covariance, and $C_1$ and $C_2$ are stabilizing constants.

For model forecast inference, the strategy applied the 15-minute model recursively, using each predicted frame as input to forecast the next 15-minute interval, proceeding iteratively until reaching a maximum lead time of 2 hours.

In the scenario simulation experiments, data from periods entirely outside the training, validation, and testing splits were employed. This configuration simulates real-world deployment scenarios in which the system ingests previously unseen satellite imagery in real time to estimate short-horizon plume evolution in the SEVIRI observation space.

### Event injection for scenario inputs

The initial cloud rise, cap–stem structure, and injection layer are assumed based on established cloud-rise formulations and observations^49,52^. The nowcasting process focuses on post-injection transport and deposition appearance in the SEVIRI Ash RGB observation space. The model ingests geostationary imagery to nowcast post-injection advection, dispersion, and bulk deposition appearance. It does not parameterize species-dependent aqueous chemistry, solubility-controlled activation, or size-resolved in-cloud and below-cloud scavenging of ^137^Cs aerosols^17,29,30^. As a result, precipitation-driven scavenging may be underrepresented or overrepresented relative to process-resolving dispersion models.

To emulate the optical signature of a localized particulate source within the final satellite composite, a synthetic plume is overlaid using a developed pixel-based algorithm. The procedure consists of the following phases:

1. Conversion of the nominal yield (in kilotons) into a pixel-quantity parameter:
  $$\text {paint\_pixels} = {\left\{ \begin{array}{ll} 1, & \text {for a yield up to }500,\text {Kt},\\ 4, & \text {for a yield up to }1{,}000, \text {Kt},\\ 8, & \text {for a yield up to }10{,}000, \text {Kt},\\ 36, & \text {for a yield of }15{,}000, \text {Kt},\\ 100, & \text {for a yield of }50{,}000, \text {Kt},\\ 169, & \text {for a yield of }100{,}000, \text {Kt}.\\ \end{array}\right. }$$
2. Determination of side-length and half-width of the sampling kernel:
  13 $$\begin{aligned} \textrm{side}&= \left\lfloor \sqrt{\text {paint\_pixels}}\right\rfloor , \end{aligned}$$
  14 $$\begin{aligned} \textrm{half}&= \left\lfloor \tfrac{\textrm{side}}{2}\right\rfloor . \end{aligned}$$
3. Transformation from geodetic coordinates $(\phi ,\lambda )$ to image coordinates $(p_x,p_y)$:
  15 $$\begin{aligned} p_x&= \textrm{round}\!\Bigl (\tfrac{\lambda - \lambda _{\min }}{\lambda _{\max }-\lambda _{\min }}\,(W-1)\Bigr ), \end{aligned}$$
  16 $$\begin{aligned} p_y&= \textrm{round}\!\Bigl (\tfrac{\phi _{\max }-\phi }{\phi _{\max }-\phi _{\min }}\,(H-1)\Bigr ). \end{aligned}$$
4. Specification of the synthetic-plume color palette (Ash-RGB convention):
  center_color = (180,20,40)    (deep red)
  inner_color = (220,50,70)    (bright red ring)
  outer_color = (80,80,80)    (dark-gray fallout zone)
5. Rendering of the plume according to the Chebyshev distance metric:
  17a $$\begin{aligned} S&= \bigl \{(dx,dy)\in \mathbb {Z}^2 \mid -\textrm{half}\le dx,dy\le \textrm{half}\bigr \}, \end{aligned}$$
  17b $$\begin{aligned} (x,y)&= (p_x + dx,\;p_y + dy), \quad 0 \le x< W,\;0 \le y < H, \end{aligned}$$
  17c $$\begin{aligned} d&= \max \!\bigl (|dx|,|dy|\bigr ), \end{aligned}$$
  17d $$\begin{aligned} C(x,y)&= {\left\{ \begin{array}{ll} \mathrm {center\_color}, & d = 0,\\ \mathrm {inner\_color}, & d = 1,\\ \mathrm {outer\_color}, & d \ge 2. \end{array}\right. } \end{aligned}$$
  where $\textrm{half}$ denotes the half-width of the square sampling kernel; *S* is the set of integer offsets within that kernel; (*x*, *y*) are the corresponding image coordinates (constrained by $0\le x<W$, $0\le y<H$); *d* is the Chebyshev distance of each offset; *C*(*x*, *y*) assigns one of the three RGB colors according to the concentric-zone rule^53^, with coloring thresholds defined in (17d).
6. Integration of the modified frame into the prediction pipeline by replacing the final historical frame, followed by model inference.

**Figure 2:**
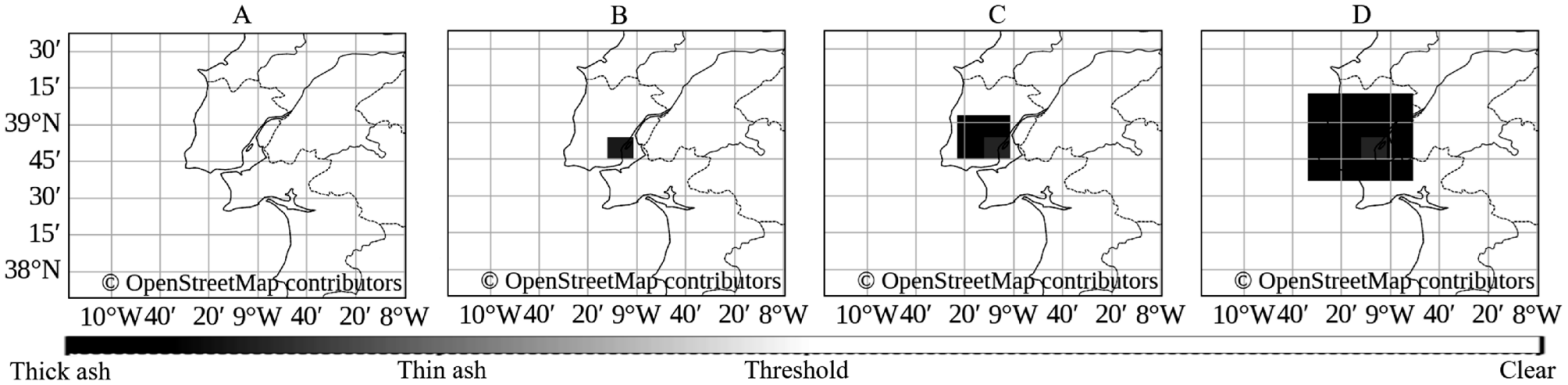
Synthetic event-injection examples over Lisbon: (**A**) no injection, (**B**) 500 kt, (**C**) 1,000 kt, (**D**) 10,000 kt. The painted pixel extent visualizes different injection sizes. Maps generated using Python 3.1 (https://www.python.org/) with OpenStreetMap contributor data (https://www.openstreetmap.org).

To illustrate the practical application of event injection for scenario inputs, Figure 2 depicts a synthetic simulation centered over the Lisbon metropolitan area. Panel A shows the original unaltered scene serving as the reference background. Panels B through D depict progressive escalation in the nominal yield parameter: a 500 kt case introduces a localized darkened region indicating moderate particulate loading; the 1,000 kt scenario expands this area substantially; and the 10,000 kt case produces an extensive plume enveloping much of western Portugal. The grayscale gradient encodes relative thickness of the synthetic ash field, with pixel values approaching zero (black color intensity) corresponding to the densest zones.

## Results and discussion

### Model optimization and performance

**Figure 3:**
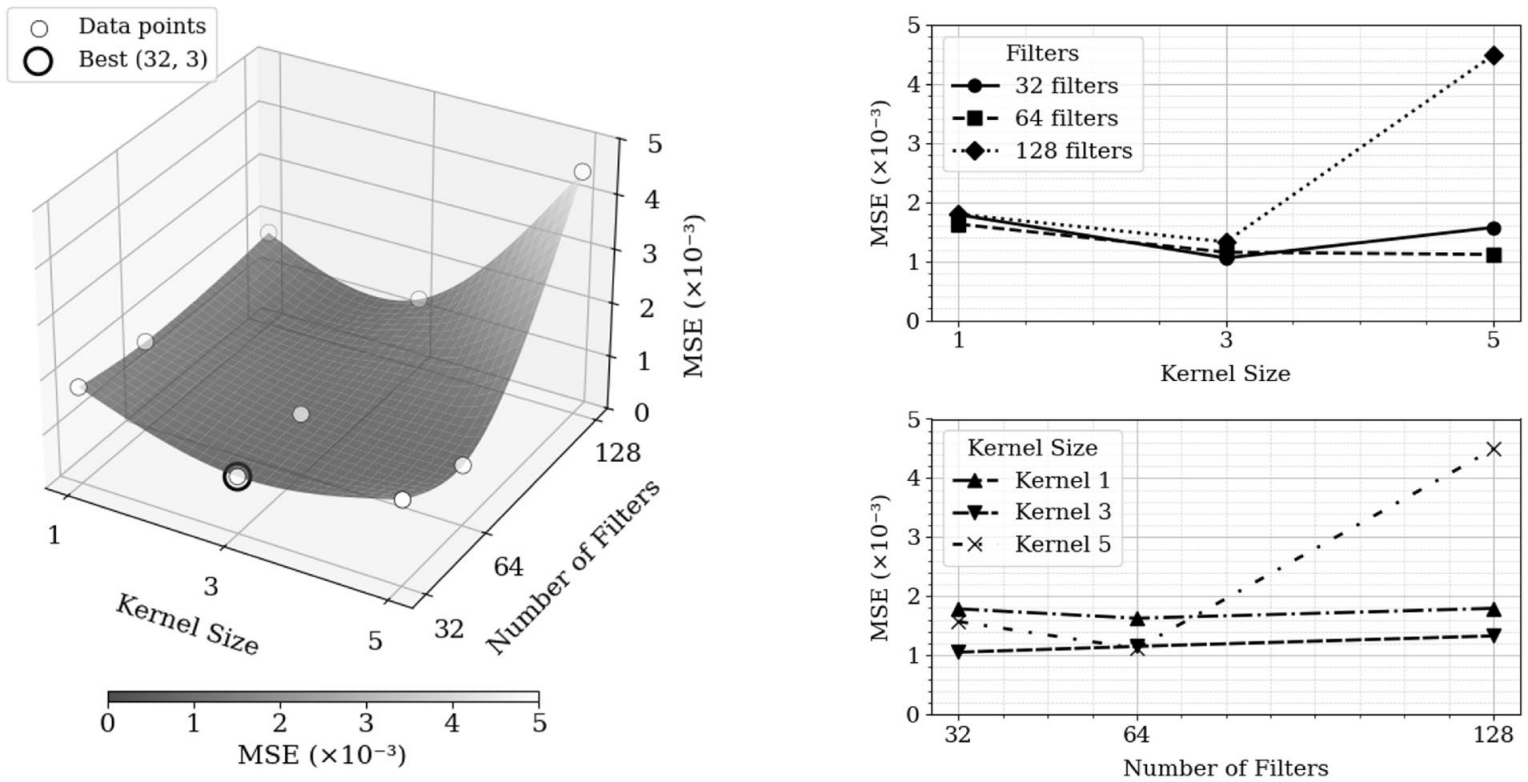
Grid search results over kernel size and number of filters for MSE optimization. Left: 3D plot with evaluated configurations, with optimal setting highlighted with a bold black ring. Right: MSE trends across kernel size (top) and number of filters (bottom) for all tested values. Surface shading represents interpolated MSE values to illustrate a possible performance landscape.

All optimization was performed using volcanic ash validation and the results of the hyperparameter grid search are presented in Figure 3. The plot visualizes the MSE across tested combinations of filter quantities and kernel sizes. The search identified that a configuration employing 32 filters and a $3\times 3$ kernel size yielded the lowest MSE. Further increases to these parameters within the search space did not produce a corresponding improvement in accuracy. Therefore, this configuration was established as the optimal hyperparameter setting and selected for the final model.

Table 2 reports performance metrics obtained using recursive forecasting at 15-minute resolution up to the 120-minute horizon on the SEVIRI volcanic ash holdout test dataset.

**Table 2:** Performance metrics (mean ± standard deviation) across different forecasting horizons using recursive forecasting with 15-minute resolution inputs (SEVIRI volcanic ash holdout test dataset).

Lead time (minutes)	MSE	MAE	RMSE	PSNR
15	0.000752 ± 0.000083	0.01947 ± 0.00113	0.02738 ± 0.00151	31.27 ± 0.48
30	0.001603 ± 0.000176	0.02826 ± 0.00171	0.03998 ± 0.00219	27.98 ± 0.48
45	0.002647 ± 0.000289	0.03669 ± 0.00224	0.05137 ± 0.00281	25.80 ± 0.48
60	0.003861 ± 0.000427	0.04494 ± 0.00274	0.06205 ± 0.00343	24.16 ± 0.48
75	0.005241 ± 0.000587	0.05306 ± 0.00313	0.07228 ± 0.00405	22.83 ± 0.49
90	0.006795 ± 0.000798	0.06112 ± 0.00357	0.08229 ± 0.00481	21.71 ± 0.51
105	0.008515 ± 0.001046	0.06911 ± 0.00401	0.09211 ± 0.00559	20.73 ± 0.52
120	0.010385 ± 0.001301	0.07700 ± 0.00445	0.10171 ± 0.00629	19.87 ± 0.53

As a proof of concept, the input steps were increased to 60-minute resolution instead of 15-minute resolution (using the same model structure and hyperparameters), with the goal of assessing if longer temporal context can lead to better forecasts at higher lead times, evaluated at 60-minute and 120-minute horizons. Table 3 compares both configurations. The results indicate that while the 60-minute step does not consistently outperform the 15-minute model at the 60-minute lead time, it demonstrates superior performance across evaluated error metrics at the 120-minute horizon. This suggests that aggregating inputs over longer temporal resolutions can enhance predictive performance for extended lead times, at the cost of temporal detail.

**Table 3:** Performance metrics (mean ± standard deviation) comparing 15-minute and 60-minute step input models at 60-minute and 120-minute lead times (SEVIRI volcanic ash holdout test dataset).

Metric	60-minute lead time		120-minute lead time	
	15-minute	60-minute	15-minute	60-minute
MSE	0.00386 ± 0.00043	0.00399 ± 0.00060	0.01039 ± 0.00130	0.00763 ± 0.00105
MAE	0.04494 ± 0.00274	0.04254 ± 0.00324	0.07700 ± 0.00445	0.06116 ± 0.00445
RMSE	0.06205 ± 0.00343	0.06301 ± 0.00473	0.10171 ± 0.00629	0.08713 ± 0.00601
SSIM	0.67167 ± 0.02126	0.64503 ± 0.02776	0.52741 ± 0.02809	0.53861 ± 0.06019
PSNR	24.1600 ± 0.48000	24.0400 ± 0.66000	19.8700 ± 0.53000	21.2200 ± 0.03000

Although the model trained with 60-minute input steps yields improved quantitative metrics at extended lead times (such as lower MSE and higher SSIM at 120 minutes), this gain comes at the cost of temporal resolution in the output. The coarser temporal sampling reduces the ability to capture fine-grained atmospheric dynamics and short-term fluctuations in plume behavior that are relevant for near-real-time operations. As a result, while the longer-step model performs better in aggregate error terms, it produces smoother, temporally less-detailed forecasts. Therefore, reducing input resolution is not preferred in this work. Likewise, increasing the input frame frequency beyond 15-minute intervals provides diminishing returns, since SEVIRI imagery is already sampled at that rate. Thus, the 15-minute step configuration offers the best balance between forecasting accuracy, temporal detail, and operational applicability.

### Scenario simulations

This section presents scenario demonstrations that use the validated volcanic ash nowcasting model together with the event-injection procedure as illustrative demonstrations. The goal is to show how the low-latency pipeline can generate short-horizon, satellite-style transport visualizations when a synthetic source is introduced into the most recent SEVIRI frame. These demonstrations are not validations of nuclear fallout plume morphology. They are image-domain scenario inputs that support rapid, consistent visualization of kinematic transport patterns in the SEVIRI observation space.

#### Urban-scale

An urban-scale scenario was produced by injecting a synthetic source (nominal 1,000 kt size parameter) centered over Berlin, Germany. The injection was initiated at 23:00 UTC on 03 July 2025, with model output generated in 15-minute increments for a total of 2 hours. The scenario is used to visualize rapid downwind spread and to illustrate how an evacuation perimeter could be overlaid based on plume extent within the 2-hour window.

**Figure 4:**
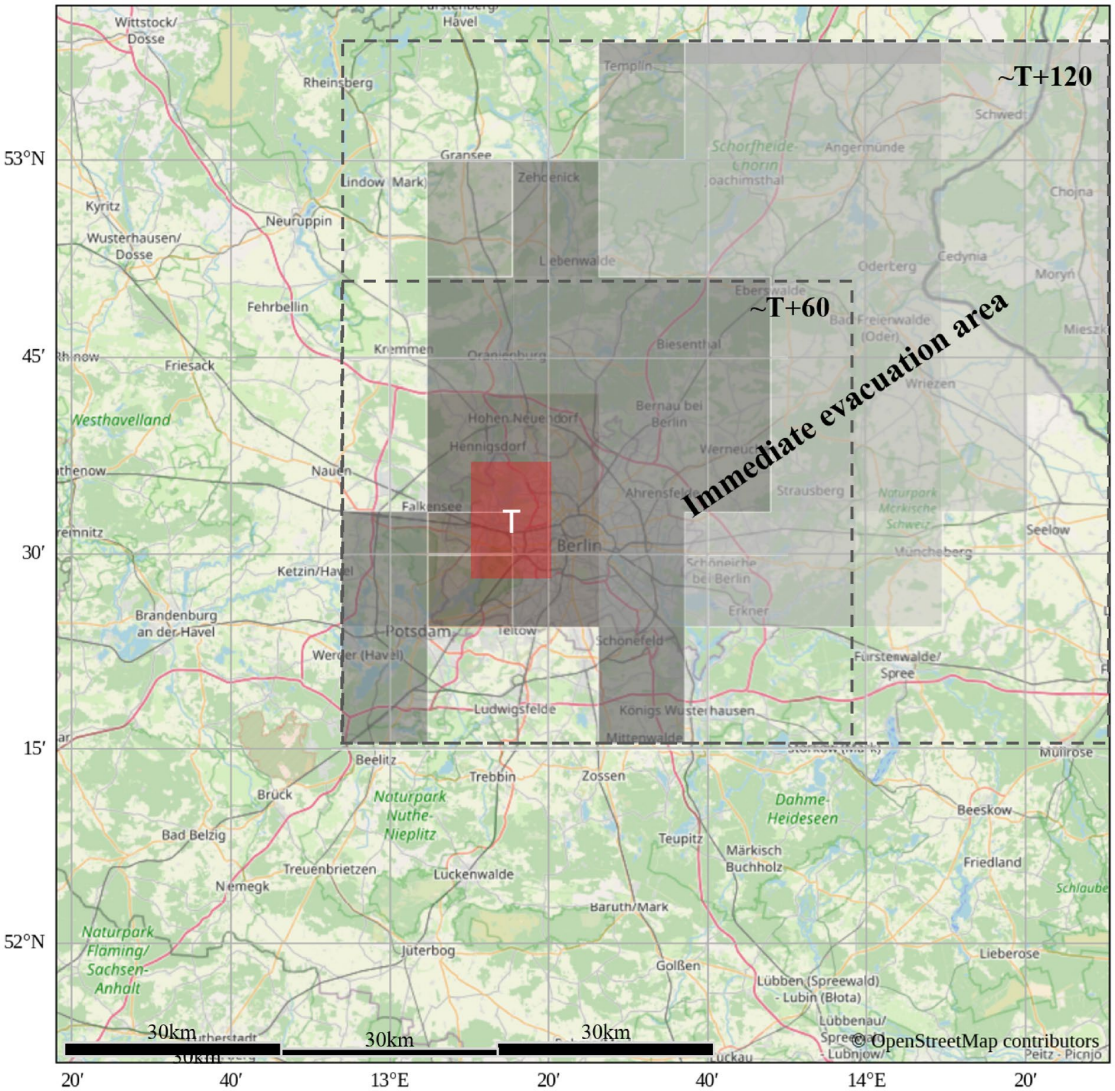
Urban-scale scenario centered over Berlin: synthetic injection with nominal 1,000 kt size parameter at 23:00 UTC on 03 July 2025. Image depicts the forecasted ash-like dispersion appearance after 2 hours and an example evacuation zone overlay based on plume footprint. Maps generated using Python 3.1 (https://www.python.org/) with OpenStreetMap contributor data (https://www.openstreetmap.org).

Figure 4 presents the injected frame and the forecasted ash-like dispersion appearance after 2 hours, with an example evacuation zone overlay derived from the modeled plume footprint.

**Figure 5:**
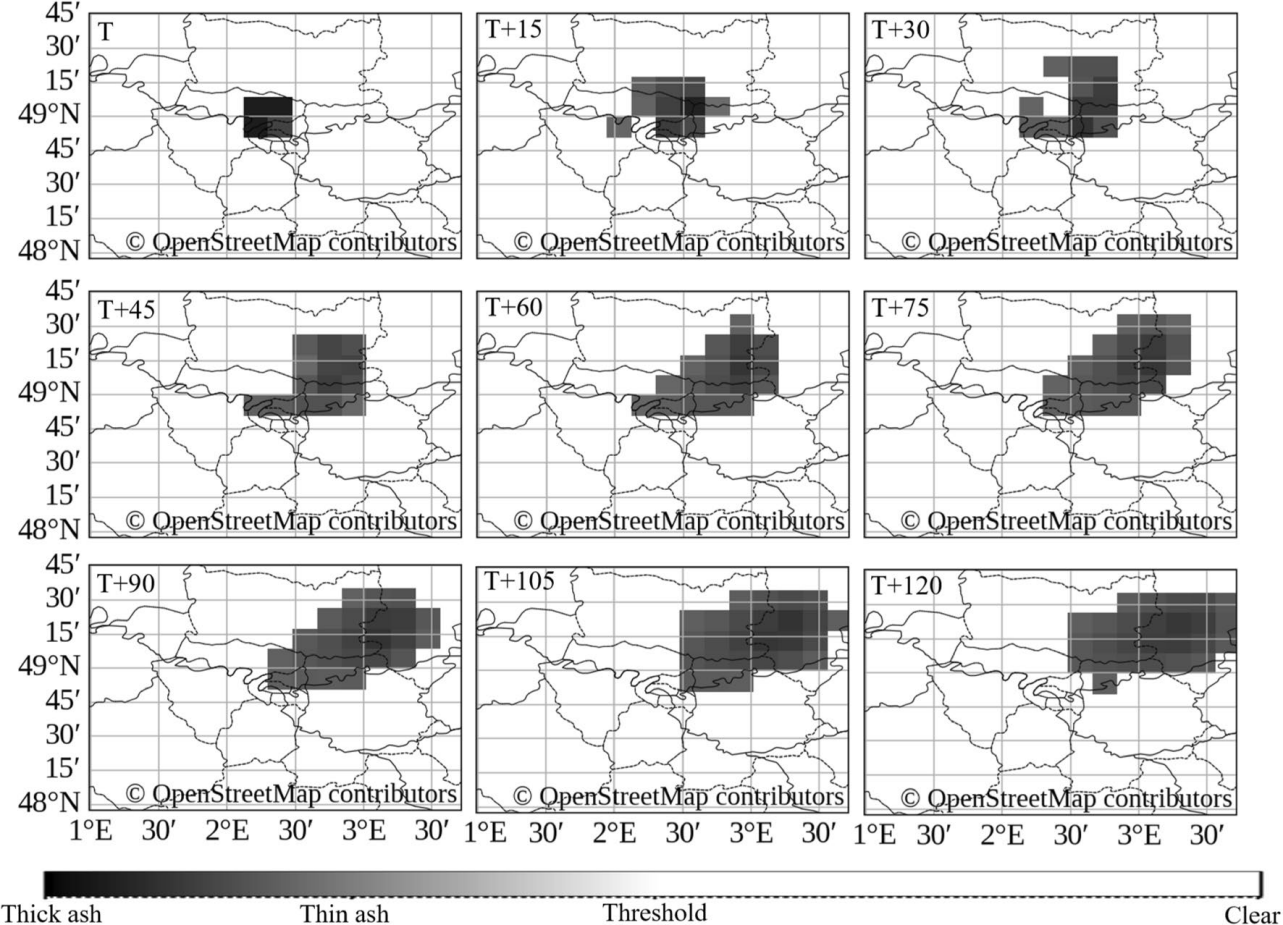
Urban-scale scenario centered over Paris: synthetic injection with nominal 1,000 kt size parameter at 00:00 UTC on 10 April 2025. The first frame shows the injected source; subsequent panels show the nowcasts (lead intervals T) produced by the volcanic ash model from the injected input sequence. Maps generated using Python 3.1 (https://www.python.org/) with OpenStreetMap contributor data (https://www.openstreetmap.org).

Expanding to a second urban setting, an additional scenario used a synthetic injection (nominal 1,000 kt) centered over Paris, France. The injection occurred at 00:00 UTC on 10 April 2025 (T), and dispersion was simulated at 15-minute intervals up to 02:00 UTC (T+120). Figure 5 showcases the injected source and subsequent nowcasts.

In Figure 5, the first 120 minutes show early-stage plume advection in the SEVIRI observation space. From frame T to T+15, the injected cloud expands radially with a clear northeastward bias, indicating prevailing winds from the southwest. By T+120, the plume becomes increasingly elongated along the northeast–southwest axis. The center of mass of the densest region shifts approximately 0.5–0.75 degrees east-northeast in longitude, corresponding to an estimated displacement of roughly 40–60 km over 120 minutes, implying a transport velocity of approximately 20–30 km/h. This pattern is consistent with typical mid-tropospheric nocturnal flow regimes over Western Europe in April^54^. These numbers describe the motion seen in the model output and do not constitute a validated estimate of radioactive concentration or deposition.

#### Country-scale

A country-scale scenario was designed with four simultaneous synthetic injections over the Iberian Peninsula, targeting Lisbon and Porto (Portugal) and Madrid and Barcelona (Spain). The nominal yield parameter was set to 10,000 kt for each injection. The injection occurred at 02:00 UTC on 26 June 2025, and the forecast horizon was 2 hours.

**Figure 6:**
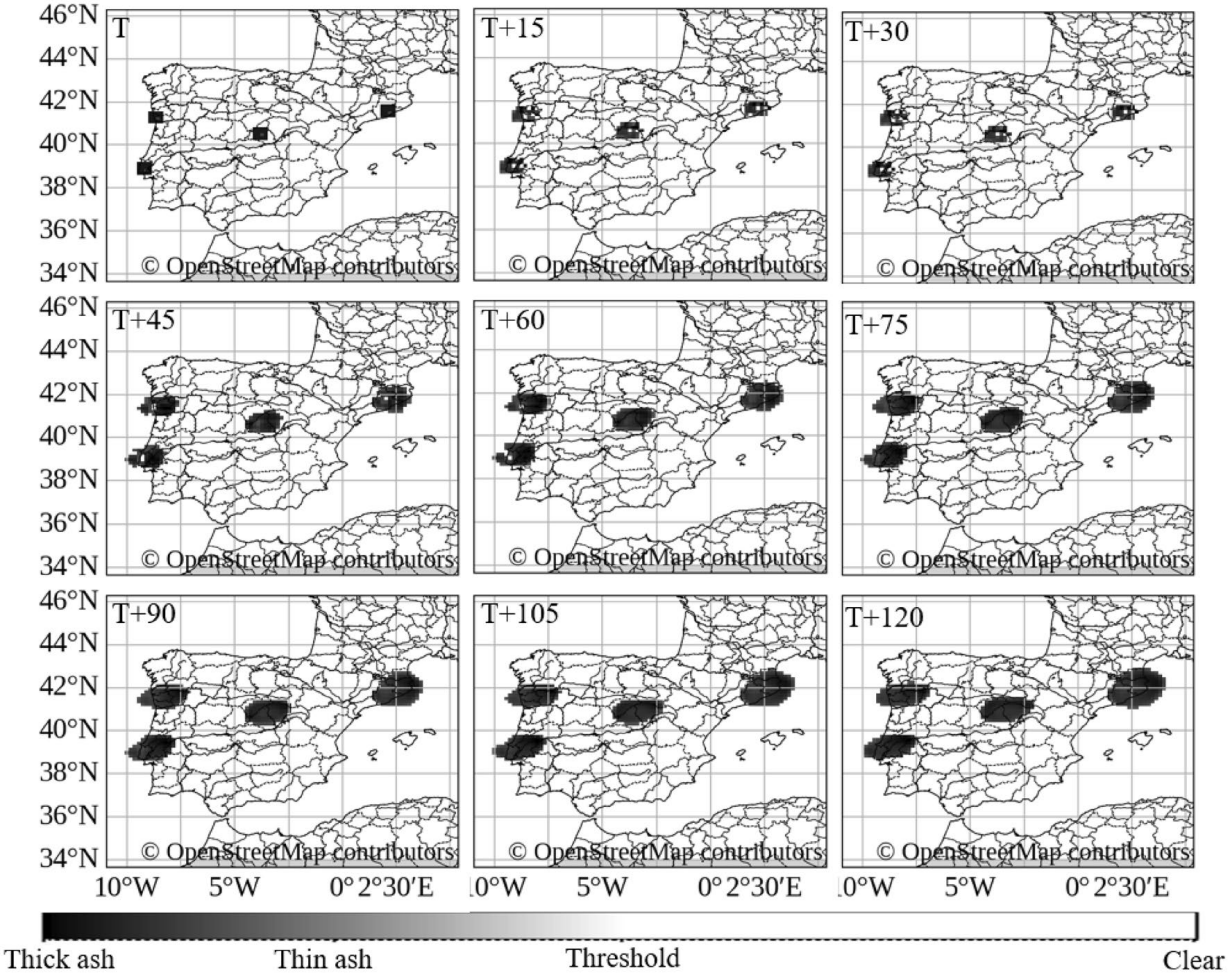
Country-scale scenario: simulated ash-like dispersion appearance from four simultaneous synthetic injections (nominal 10,000 kt size parameter) over Lisbon, Porto, Madrid, and Barcelona at 02:00 UTC on 26 June 2025. Maps generated using Python 3.1 (https://www.python.org/) with OpenStreetMap contributor data (https://www.openstreetmap.org).

Figure 6 depicts the resulting ash-like dispersion appearance from the four simultaneous injections. At time T, the injected clouds are localized over each target area, then expand and begin to merge. By T+90 to T+120, large portions of both Portugal and Spain are affected in the image-domain visualization. The color gradient from black (dense) to white (clear sky) indicates varying synthetic concentrations. This scenario illustrates how multiple sources can be handled within the same low-latency workflow.

#### Continental-scale

A continental-scale scenario injected synthetic sources (nominal 10,000 kt) at 18 major European capitals: Paris, London, Rome, Berlin, Madrid, Lisbon, Vienna, Amsterdam, Brussels, Athens, Prague, Warsaw, Budapest, Stockholm, Copenhagen, Dublin, Oslo, and Helsinki. The objective was to evaluate whether the system can handle many simultaneous injections while producing satellite-style imagery up to a 2-hour forecast horizon.

**Figure 7:**
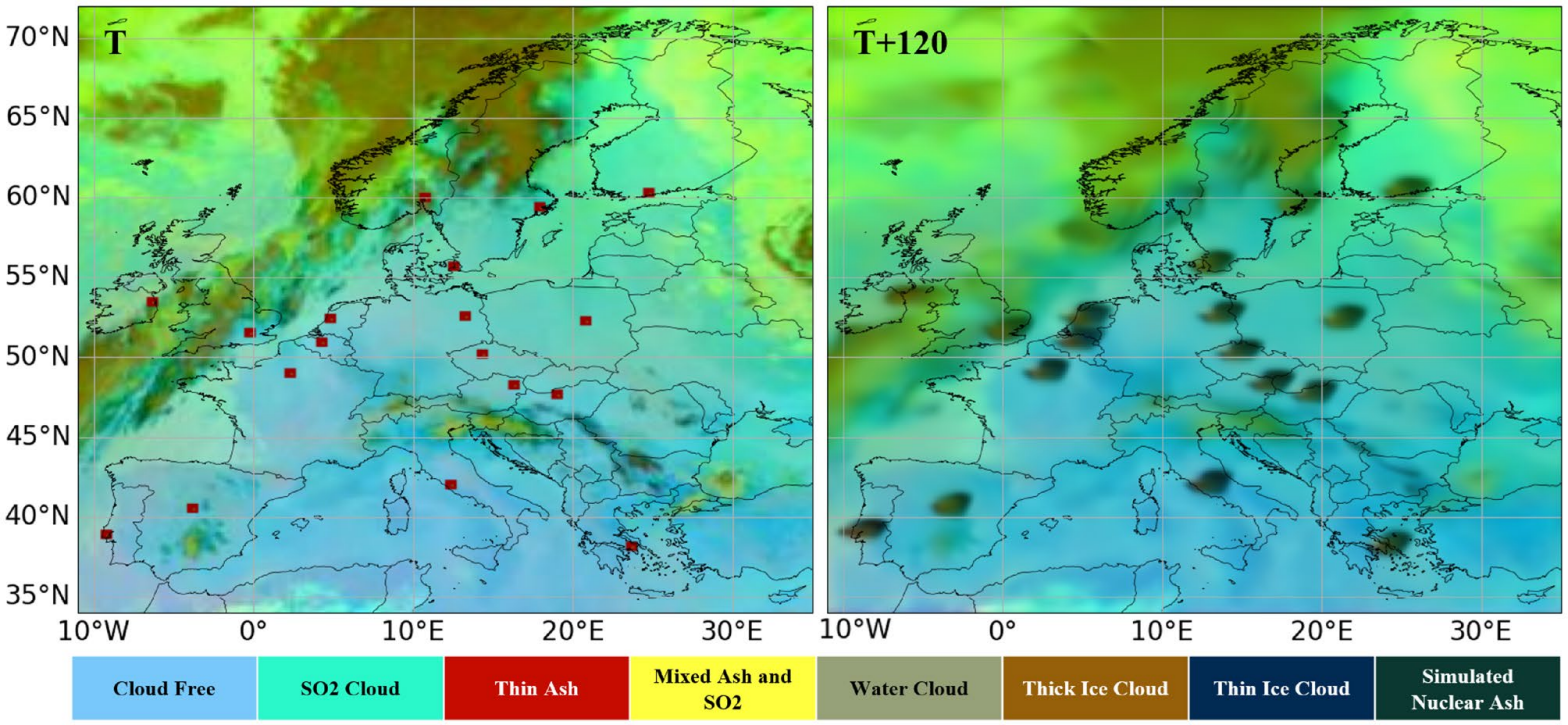
Continental-scale scenario: synthetic high-resolution satellite-style visualization of 18 simultaneous injections across Europe, initialized at 06:00 UTC on 01 July 2025. Maps generated using Python 3.1 (https://www.python.org/) with OpenStreetMap contributor data (https://www.openstreetmap.org).

Figure 7 presents the results. The left panel (T) shows the baseline SEVIRI Ash RGB product with red squares marking injection locations. The right panel (T+120) presents the model-generated forecast showing the expected ash-like dispersion appearance two hours post-injection. Color classification is adapted from EUMETSAT documentation.

**Figure 8:**
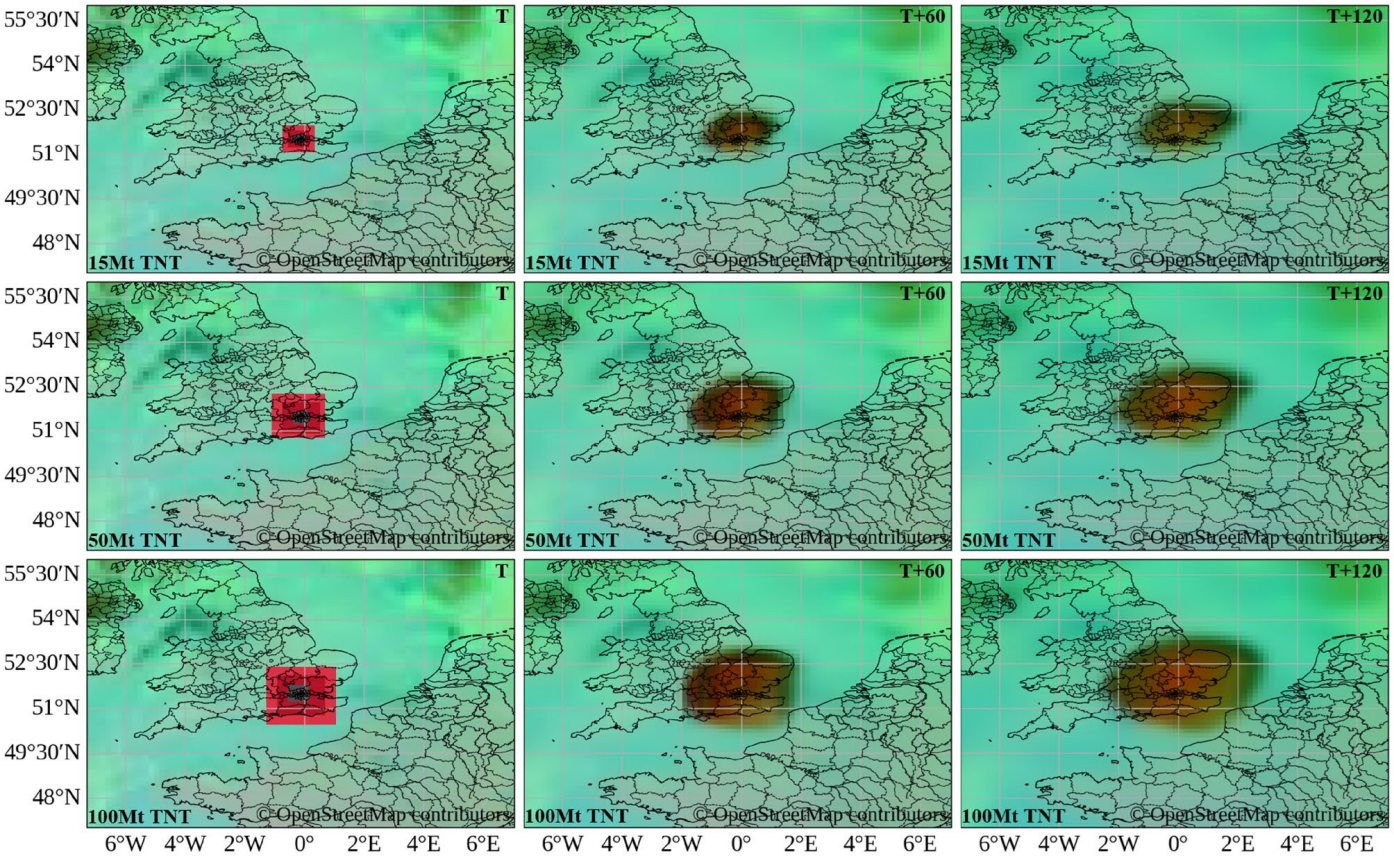
Urban-scale size-parameter scenarios over London: synthetic injections with nominal yields of 15, 50, and 100 Mt, initiated at 03:00 UTC on 10 July 2025. Each row shows a different nominal yield; columns represent detonation time (T), 60 minutes (T+60), and 120 minutes (T+120). Fallout-affected regions are highlighted in brown. Maps generated using Python 3.1 (https://www.python.org/) with OpenStreetMap contributor data (https://www.openstreetmap.org).

To explore upper-bound size parameters in an urban setting, additional scenarios were generated over London using nominal yields of 15 Mt, 50 Mt, and 100 Mt. Figure 8 shows that the footprint grows nonlinearly with the nominal yield parameter and that, at the largest value, the plume crosses the English Channel in the image-domain visualization. These outputs illustrate how the injection size ladder affects the apparent plume extent under the same background meteorology and model dynamics, without constituting validation for nuclear fallout physics.

### Relation to process-resolving numerical models

Regional intercomparison studies of Fukushima ^137^Cs demonstrate that Eulerian and Lagrangian models driven by identical meteorological fields and source terms can reproduce observed concentration peaks and deposition patterns. Model performance depends mainly on the treatment of wet scavenging and deposition processes, as well as on grid spacing of approximately 1–3 km^41,42^. Urban- and local-scale systems such as SWIFT–RIMPUFF and Micro-SWIFT–SPRAY (MSS) further capture building-induced flow and street-canyon circulation, validated through wind-tunnel experiments^43^.

The present framework introduces distinct trade-offs. It focuses on the kinematic evolution of plumes detected (or injected) in the SEVIRI observation space, representing advection and dispersion patterns without explicitly resolving microphysical processes such as in-cloud and below-cloud scavenging, hygroscopic activation, or flow modification by buildings. This simplification can introduce bias in strongly precipitating conditions dominated by aqueous scavenging and in dense urban environments. Spatial and temporal resolution are inherited directly from SEVIRI observations, with a nominal sampling of about 3 km at nadir and a 15 min revisit time, which is coarser than the 1 km resolution of typical numerical weather prediction models or the meter-scale detail of CFD frameworks.

The system operates without the need for NWP preprocessing, source inversion, or explicit urban geometry. On Jetson AGX Orin hardware, the complete workflow, including data download and inference, required less than five seconds per 15 min interval, allowing updates synchronized with each SEVIRI acquisition. The framework is designed as an observation-driven nowcasting layer that provides rapid situational awareness and prioritization support while more complex coupled or high-resolution models are initialized and assimilate new data.

The resulting concentration and deposition fields appear coarser than those produced by regional intercomparison studies or urban-scale CFD and Lagrangian simulations. This reflects an explicit trade-off between detailed process fidelity and computational efficiency, robustness to incomplete input data, and suitability for edge deployment.

Quantitative comparison with process-resolving systems such as HYSPLIT or WRF-Chem would require outputs harmonized in domain, cadence, and projection with SEVIRI imagery. Such matched datasets are not publicly available for the 2020–2025 period. The present study therefore focuses on observation-based validation against unseen SEVIRI volcanic ash sequences, which is the appropriate benchmark for a computer-vision nowcasting system. Scenario simulations presented here complement, rather than replace, future numerical intercomparisons when compatible datasets become accessible.

## Conclusion

This work addresses the need for rapid hazard assessment by developing and validating a low-latency deep learning framework for near-real-time nowcasting of volcanic ash dispersion from SEVIRI Ash RGB imagery. A ConvLSTM network trained on an extensive satellite archive predicts plume evolution up to two hours ahead using 15-minute recursive nowcasts, achieving a MAE of 0.019 and an SSIM of 0.88 for 15-minute forecasts on the SEVIRI volcanic ash holdout test dataset. The implementation sustains sub-five-second end-to-end latency on an NVIDIA Jetson AGX Orin, including data acquisition and inference, supporting operational deployment synchronized with SEVIRI updates.

In addition to volcanic ash validation, this study introduces a general event-injection framework that overlays synthetic plumes of varying sizes into the latest satellite frame to create scenario inputs for the nowcasting pipeline. Demonstrations parameterized by nuclear-yield-inspired sizes at urban, national, and continental scales illustrate how the framework can support rapid, satellite-style scenario visualization of kinematic transport patterns. These scenario outputs are not validated nuclear fallout predictions and do not model source-term physics, early-time plume rise thermodynamics, or radionuclide-specific transformation and scavenging.

Limitations define future directions. Using volcanic ash as the supervised target and employing heuristic event injection are necessary simplifications. Future work could train with synthetic outputs from high-fidelity dispersion simulations (e.g., FALL3D or HYSPLIT) to better capture source-specific properties for other particulate classes, and could integrate real-time meteorological fields to move toward a more physically informed forecasting engine. Validation in this study used an independent SEVIRI test dataset rather than historical event reconstructions. Public, standardized datasets with sufficient spatiotemporal alignment to SEVIRI are not available for direct supervision of radionuclide plumes in the 2020–2025 period. Consequently, validation focused on quantitative performance against unseen volcanic ash imagery, while scenario demonstrations were presented as illustrative applications of the general pipeline.

## Data Availability

The code supporting this study is publicly available on Zenodo at: https://doi.org/10.5281/zenodo.18430542
